# An EAAT2b/SLC1A2b-mediated chloride leak current enables rapid cone photoreceptor signalling

**DOI:** 10.1098/rsob.250347

**Published:** 2025-11-26

**Authors:** Jingjing Zang, Stephanie Niklaus, Stephan C. F. Neuhauss

**Affiliations:** ^1^ Department of Molecular Life Sciences, University of Zurich, Zürich, Switzerland; ^2^ Institute of Veterinary Physiology, University of Zurich, Zürich, Switzerland

**Keywords:** vision, photoreceptor, excitatory amino acid transporter, anion current, zebrafish, genome duplication

## Abstract

Excitatory amino acid transporters not only mediate high-affinity glutamate uptake but also conduct an uncoupled chloride current. In zebrafish, a whole-genome duplication gave rise to two *eaat2* paralogues with distinct roles. Excitatory amino acid transporter 2a (SLC1A2b, GLT-1) functions primarily in Müller glia as a glutamate transporter, whereas excitatory amino acid transporter 2b is expressed in cone photoreceptors and exhibits a prominent glutamate-independent chloride current. We hypothesized that this leak current stabilizes the cone resting membrane potential, thereby supporting rapid visual signalling. In order to test this hypothesis, we generated *eaat2b* knockout zebrafish using CRISPR-Cas9-mediated genome editing. While *eaat2b* mutants showed no gross morphological abnormalities, they exhibited reduced electroretinogram b-wave amplitudes. Consistent with our hypothesis, *eaat2b*-deficient larvae displayed a significant reduction in flicker fusion electroretinogram power at each stimulus frequency, indicating impaired temporal processing likely due to delayed repolarization of cone photoreceptors. Our findings reveal a critical role for an excitatory amino acid transporter 2b-mediated chloride anion leak current in regulating the kinetics of photoreceptor responses. This functional innovation, enabled by a whole-genome duplication in the teleost lineage, highlights how gene duplications can lead to the acquisition of physiologically relevant new functions.

**Figure:**
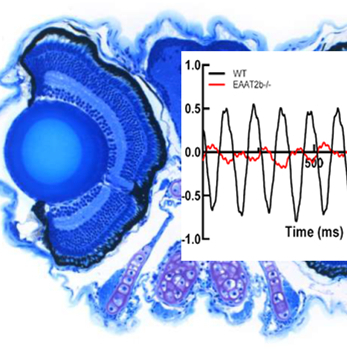

## 1. Introduction

Excitatory amino acid transporters (EAATs) are high-affinity glutamate transporters that tightly regulate extracellular glutamate concentrations in the vertebrate central nervous system, including the retina. Glutamate uptake by EAATs is an electrogenic process driven by the co-transport of three sodium ions and one proton, coupled with the counter-transport of one potassium ion [1]. In addition to this coupled transport cycle, EAATs also mediate a thermodynamically uncoupled anion conductance, primarily carried by chloride ions. This anion current, which varies in magnitude across EAAT subtypes, affects membrane potentials, typically causing hyperpolarization in the adult nervous system. In the retina, this unconventional feature of EAATs is harnessed to inhibit ON bipolar cells, thereby transforming glutamate, classically an excitatory neurotransmitter, into an inhibitory signal [2–4]. Similar roles for EAAT-mediated inhibition have recently been reported in other regions of the brain [5].

In most vertebrate retinas, glutamate clearance is primarily mediated by EAAT2 located on Müller glia cells [4,6–9]. Due to an ancient whole-genome duplication event in the teleost lineage, zebrafish possess two EAAT2 ohnologues—*eaat2a* and *eaat2b*—that have undergone subfunctionalization [8,10]. While EAAT2a is localized to Müller glial cells and primarily mediates glutamate uptake, EAAT2b is expressed presynaptically in cone photoreceptors [9]. Notably, these two isoforms differ not only in their cellular localization but also in their biophysical properties: EAAT2a exhibits minimal anion conductance, whereas EAAT2b displays a prominent leak current in the absence of glutamate [9]. This observation led to the intriguing hypothesis that EAAT2b’s principal physiological role is not glutamate clearance, but rather stabilization of the cone photoreceptor resting membrane potential in darkness. Given that the chloride reversal potential closely matches the dark resting potential of cones [11], the chloride leak through EAAT2b would oppose any deviation in membrane voltage, thereby effectively acting as a voltage clamp.

To test this hypothesis, we generated a stable eaat2b mutant zebrafish line and assessed photoreceptor function using electroretinography (ERG). Mutants displayed markedly reduced light-evoked responses and delayed recovery in a flicker fusion paradigm. These results are consistent with the proposed role of EAAT2b in shaping the fast, transient responses of cone photoreceptors and provide a mechanistic basis for earlier suggestions that EAAT2b contributes to temporal resolution in cone vision [12].

## 2. Results

In a previous work, we have shown that the zebrafish genome contains two *eaat2* ohnologues (paralogues originated from a whole-genome duplication) that show divergent expression patterns in the retina with *eaat2a* transcripts primarily found in Müller glia cells, whereas *eaat2b* RNA is most abundant in cone photoreceptors [8,9]. EAAT2b protein is localized to cone pedicles (cone synapses), but absent in spherules (rod synapses) [9]. In contrast to *eaat2a* knockdown animals, *eaat2b* knockout larvae showed only a mild impairment in visual function assayed by the ERG, potentially attributed to an insufficient reduction in EAAT2b protein levels by the morpholino injection [9].

**Figure 1.**
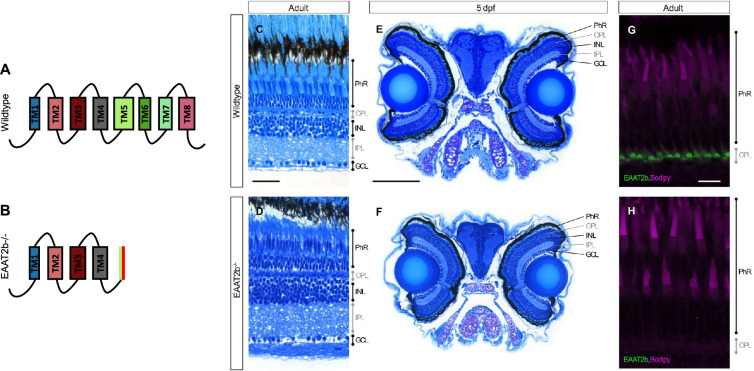
Characterization of *eaat2b* mutant zebrafish. The mutation causes a deletion that results in a premature stop codon, leading to a predicted truncated EAAT2b protein (A,B). Retinal histological sections from adult wild-type (C), adult *eaat2b*^−/−^ (D), 5 dpf wild-type (E) and 5 dpf *eaat2b*^−/−^ (F) zebrafish show no apparent morphological abnormalities in either larval or adult eyes. Immunofluorescence staining for EAAT2b (green) and Bodipy (magenta) is shown in adult wild-type (G) and *eaat2b* ^*−*/−^ (H) retinas. GCL, ganglion cell layer; INL, inner nuclear layer; IPL, inner plexiform layer; OPL, outer plexiform layer; PhR, photoreceptors; TM, transmembrane domain.

To address this limitation, we have generated an *eaat2b* knockout line using CRISPR-Cas9 genome editing (electronic supplementary material, figure S1A). The mutation introduces a deletion causing a premature stop codon, resulting in a predicted truncated EAAT2b protein (figure 1A and electronic supplementary material, figure S1B). In *eaat2b* mutant eyes, EAAT2b immunofluorescence is absent, indicating loss of protein expression (figure 1G,H). Despite this, both larval and adult mutant eyes show no detectable morphological abnormalities (figure 1C–F).

**Figure 2.**
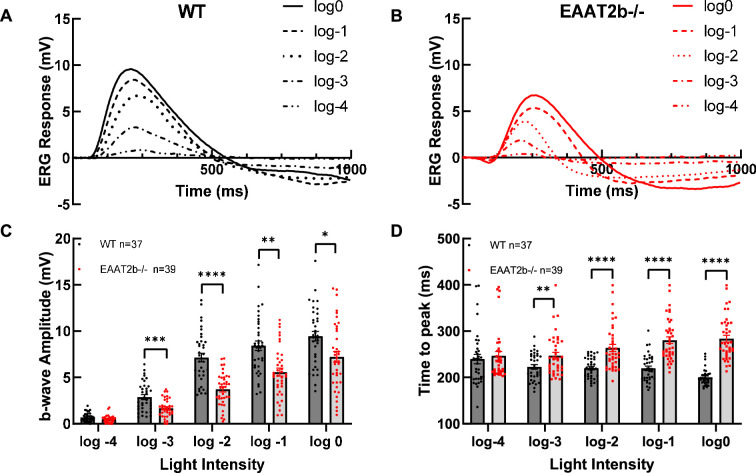
*eaat2b* mutant zebrafish larvae exhibit reduced ERG b-wave amplitude and impaired response kinetics. Representative ERG responses of WT controls (A) and *eaat2b* mutants (B) at different flash intensities. (C) Quantification of the b-wave amplitude across five different light intensities. *eaat2b*^−/−^ larvae (in red) show a significant reduction in ERG b-wave amplitudes compared to WT controls (in black). Statistical significance: *p* = 0.0142 at log 0, *p* = 0.0013 at log −1, *p* < 0.0001 at log −2, *p* = 0.0005 at log −3 and *p* = 0.0771 at log −4. In addition, the time to peak was quantified (D). Statistical significance of time to peak: *p* = 0.6161 at log −4, *p* = 0.0074 at log −3, *p* < 0.0001 at log −2, *p* < 0.0001 at log −1 and *p* < 0.0001 at log 0. Statistical analysis was performed using *t*-tests and nonparametric tests in GraphPad Prism, v. 8. Data are presented as mean ± s.e.m. Significance levels are indicated as follows: *p < 0.05; **p < 0.01; ***p < 0.001; ****p < 0.0001.

To investigate the functional role of EAAT2b in the zebrafish retina, we performed white light ERG on 5 days post-fertilization (dpf) larvae across different light levels. ERG records the summation of field potentials generated by the entire retina in response to light stimuli. In a typical zebrafish wild-type (WT) larval ERG response (figure 2A), the small negative a-wave, representing photoreceptor hyperpolarization in response to light, is often masked by the much larger positive b-wave, reflecting the depolarization of ON bipolar cells. At 5 dpf, rod responses contribute little to nothing to the ERG; hence, the recorded light responses at this stage are exclusively cone-driven [13–16]. Any potential weak contributions of rods to the dark-adapted ERG would also be diminished by the light-adapted state of the larvae [17,18].

Fish homozygous for *eaat2b* null alleles exhibited a marked reduction in ERG b-wave amplitude across all light intensities, except for the lowest intensity, where it is probably masked by the proximity to background noise (figure 2B,C). At a light intensity of log −2, the b-wave amplitude in mutant fish was reduced by approximately 50% (figure 2C), and the time to reach the b-wave peak was significantly extended (figure 2D). At the highest light intensity, the mutant response took 30% longer to reach peak amplitude.

**Figure 3.**
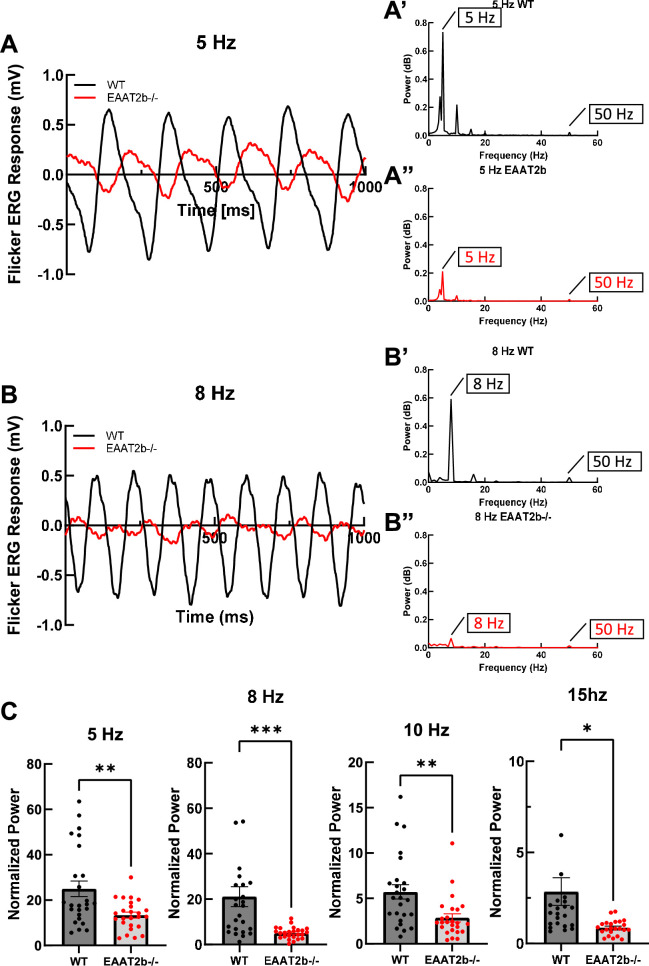
Temporal resolution of flicker ERG responses is reduced in *eaat2b* mutant fish. (A) Representative flicker ERG responses to a 5 Hz stimulus in WT controls (black) and *eaat2b*^−/−^ larvae (red). Corresponding FFT power plots for WT (A′) and *eaat2b*^−/−^ (A*″*) were generated in MATLAB from the traces shown in (A). (B) Representative flicker ERG responses to an 8 Hz stimulus in WT controls (black) and *eaat2b*^−/−^ larvae (red). Corresponding FFT power plots for WT (B′) and *eaat2b*^−/−^ (B*″*) were generated from the traces shown in (B). (C) Normalized power of flicker ERG responses at specified frequencies in WT controls and *eaat2b*^−/−^ larvae. Power values were normalized to the 50 Hz line-noise peak. Statistical significance: *p* = 0.0039 at 5 Hz, *p* = 0.0007 at 8 Hz, *p* = 0.0031 at 10 Hz, *p* = 0.0133 at 15 Hz. Statistical analysis was performed using *t*-tests and nonparametric tests in GraphPad Prism, v. 8. Data are presented as mean ± s.e.m. Significance levels are indicated as follows: *p < 0.05; **p < 0.01; ***p < 0.001.

In a previous study, we reported a large leak current of EAAT2b in oocyte recordings, meaning that even in the absence of the ligand glutamate, EAAT2b supports a current flow [9]. Since the expected reversal potential of chloride is very close to the dark resting potential of photoreceptors [19,20], this leak current could function as a clamp that would impact the kinetic properties of the light response. In order to test this hypothesis, we recorded ERG responses to flickering stimuli at different frequencies (5, 8, 10 and 15 Hz). We used a fast Fourier transform (FFT) algorithm in MATLAB to extract the power at each stimulus frequency (figure 3A*–*A″*,*B*–*B″), which was then normalized against the power at 50 Hz (line noise). The normalized power at each stimulus frequency was significantly reduced in *eaat2b*^−/−^ fish compared to WT controls (figure 3C), indicating that cone-mediated visual temporal resolution is indeed compromised in the absence of EAAT2b protein.

## 3. Discussion

EAATs are remarkable in their dual functionality, acting both as high-affinity glutamate transporters and as anion-selective channels, with current amplitudes that vary significantly across different EAAT proteins [21–23]. These two functions are thermodynamically uncoupled and may therefore adapt differently under selective pressure, as seen here for zebrafish *eaat2* ohnologues. These two ohnologues originate from the whole-genome duplication (R3) early in the teleost lineage [10]. The ancestral EAAT2 protein likely functioned mainly as a transporter with small anion current amplitudes, similar to extant vertebrate EAAT2 proteins. This enabled the ohnologue EAAT2b to develop another physiological function, acquiring both a divergent expression pattern and biophysical properties. EAAT2b is expressed in cone photoreceptors, while still retaining low remaining expression in Müller glia cells [9]. This spatial diversification is also mirrored in their biophysical properties. EAAT2a features a low anion conductance, in line with its primary function as a glutamate transporter, whereas EAAT2b displays a low transporter capacity but a larger anion conductance [9]. Interestingly, EAAT2b exhibits the anion conductance in the absence of glutamate binding, referred to as a leak current.

In a previous study, we generated knockdown zebrafish larvae to further our understanding of the physiological function of EAAT2b. We reported that larvae with reduced EAAT2b protein levels presented a small reduction in their response to white light ERG [9].

Here, we report the generation of a genomic *eaat2b* knockout. In line with their predominant expression in the visual system, we did not observe any increase in lethality. However, we found a larger reduction of light responsiveness in the ERG, arguing for a complete absence of EAAT2b protein in mutant animals in contrast to an incomplete depletion in the knockdown animals.

What could the physiological function of a leak current for chloride ions in cone photoreceptors be?

The direction of chloride (the relevant ion in the outer retina) flow depends on the reversal potential of cone photoreceptors for chloride. At this reversal potential, there is no net flow of chloride ions across the membrane, since inward and outward flow rates are matched. For technical reasons, we did not succeed in directly recording the chloride reversal potential in tiny larval zebrafish cones, but previous studies in other species indicated that the dark resting potential of photoreceptors is very close to the chloride reversal potential [19,20]. Hence, a large leak current of chloride ions would stabilize (clamp) the resting potential. Any deviation will be counteracted by chloride flow, thereby stabilizing the resting potential and enabling a faster return to resting potentials after perturbations. Hence, the loss of such a leak current in the mutant animals predicts a difference in temporal aspects of vision, due to the increased time to return to the resting potential. We tested this prediction by determining the flicker fusion ERG power at each stimulus frequency, with lower power reflecting reduced temporal resolution. In line with our hypothesis, there was a marked decrease in the flicker fusion ERG power in animals lacking EAAT2b, indicating that the reported leak current indeed affects kinetic properties of photoreceptors, presumably by delaying the return to resting potentials.

In sum, our study uncovered a crucial role of a chloride leak current carried by the EAAT2b protein in regulating the kinetics of visual perception at the level of photoreceptors. This functional shift of the EAAT2 protein was enabled by a whole-genome duplication event in the teleost lineage, which allowed the acquisition of novel functions.

## 4. Material and methods

### 4.1. Fish maintenance

Zebrafish (*Danio rerio*) of the Wik strain were maintained under standard conditions in a 14 h light/10 h dark cycle, as described by Mullins *et al.* [24]. Larvae were raised in E3 embryo medium (5 mM NaCl, 0.17 mM KCl, 0.33 mM CaCl₂, 0.33 mM MgSO₄ and 10⁻⁵% methylene blue) at 28°C. The light intensity inside the incubator (Memmert HPP10), where the larvae were reared, was approximately 1570 lux, as measured with a digital light meter (PeakTech 5020). All experiments were conducted in accordance with the ARVO Statement for the Use of Animals in Ophthalmic and Vision Research and were approved by local authorities (Veterinäramt Zürich TV4206).

### 4.2. Generation of CRISPR/Cas9 knockout line

Stable homozygous fish carrying *eaat2b* mutant alleles were generated using the CRISPR/Cas9 approach. Target sites were selected by using the https://www.zifit.partners.org and https://chopchop.rc.fas.harvard.edu/ target site prediction tools. Possible targets were further tested for potential off-target sites and had to meet the requirement of a 5′ GG, which enables T7-mediated *in vitro* transcription. The 5′ GG is followed by 18 gene-specific nucleotides and a 3′ NGG palindromic adjacent motif (GG-N18-NGG). Single-guide RNA (sgRNA) synthesis was carried out with a polymerase chain reaction-based approach. To enhance the mutagenesis rate, two targets were chosen, lying 39 nucleotides apart. Forward primers of the sequence GAAATTAATACGACTCACTATAGGN 18GTTTTAGAGCTAGAAATAGC together with the common, partially overlapping reverse primer AAAAGCACCGACTCGGTGCCACTTTTTCAAGTTGATAACGGACTAGCCTTATTTTAACTTGCTATTTCTAGCTCTAAAAC were used for dsDNA synthesis using a high-fidelity Phusion polymerase (New England Biolabs). These target-specific amplicons served as templates for T7 *in vitro* transcription (MEGAshortscrip T7 Transcription Kit, Ambion). sgRNAs were purified using a Megaclear Kit (Ambion), followed by an ethanol precipitation. Genomic target sequences used were as follows: *eaat2b* (target 1): GGGAGAGAAGGCCAAACTGA (cDNA position 717–736) and *eaat2b* (target 2): GGCTTGTCGGCATGATCATG (cDNA position 776–795). Protein/sgRNA complex formation of the two targets was carried out in separate tubes for 10 min at 37°C. Each mix contained 150 ng μl^−1^ sgRNA, 814 ng μl^−1^ Cas9 protein (kindly provided by Darren Gilmour) and 300 mM KCl. The final injection mix consisted of a 1 : 1 dilution of the two mixes. One nanolitre was injected into the cell of the one-cell stage embryo. This approach introduced a 7 bp deletion and 40 bp insertion at the first target site and a 10 bp deletion and 60 bp insertion at the second site, resulting in an overall 83 bp frameshift mutation (genotyping primer: s GCGGCATGAATGTGTTAG; as TTCATGTCCTTTCCGGGT). No off-target sites have been predicted by a bioinformatic algorithm [25].

### 4.3. Histology

Whole larvae or adult eyes were fixed in 4% paraformaldehyde overnight at 4°C. Larvae were subsequently dehydrated through a graded ethanol series in PBS (50, 70, 80, 90, 95 and 100% ethanol). After dehydration, larvae were immersed in a 1 : 1 mixture of ethanol and Technovit 7100 (Heraeus Kulzer, Germany) containing 1% Hardener I for 1 h, then transferred to 100% Technovit for overnight incubation at room temperature. For embedding, the samples were placed in Technovit 7100 polymerization medium within plastic moulds and dried at 37°C for 1 h. Thin sections of 3 μm were obtained using a microtome and subsequently mounted onto slides before drying at 60°C. Staining was performed with Richardson’s solution (Romeis) comprising 0.5% Borax, 0.5% Azur II and 0.5% methylene blue, and slides were mounted with Entellan (Merck, Germany). Brightfield images were captured with an Olympus BX61 microscope.

### 4.4. Immunohistochemistry

Immunohistochemistry was performed as previously described [9]. Sections were incubated with a custom-made guinea pig anti-EAAT2b primary antibody (1 : 100) [9], followed by a goat anti–guinea pig Alexa Fluor 488 secondary antibody (Invitrogen; 1 : 1000 in PBS). For counterstaining, Bodipy TR Methyl Ester (Invitrogen) was applied at 1 : 300 dilution in PDT buffer (PBS containing 1% DMSO and 0.1% Triton X-100) for 20 min following secondary antibody washes. Images were acquired using a Leica SP5 confocal microscope. Image processing was performed with Imaris software (Bitplane).

### 4.5. White light electroretinography

White light ERG recordings were conducted on 5 dpf knockout and WT control larvae, following the protocol described by Zang *et al*. [26]. Briefly, larvae were dark-adapted for at least 30 min, and all preparatory steps prior to recording were carried out under dim red light. The larval eye was removed and placed on a 1.5% agarose plate, with a chlorided silver wire inserted to serve as the reference electrode. The recording electrode (GB100F-10, Science Products) was positioned at the centre of the cornea. An HPX-2000 xenon light source (Ocean Optics) was used to deliver 100 ms light stimuli at five different intensities (log −4 to log 0), with a 10 s interval between each stimulus. Its light spectrum was provided in Zang *et al.* [26]. The b-wave amplitude, b-wave time to peak and b-wave recovery half-life were quantified using MATLAB (R2023b). Figures were generated, and statistical analyses were performed using GraphPad Prism 9 (GraphPad Software, San Diego, CA, USA).

### 4.6. Flicker fusion electroretinography

Flicker fusion ERGs were measured as previously described [26]. A white LED light source (Ocean Optics, LSM series) controlled by an LDC-1 controller (Ocean Optics) was used. Its light spectrum was provided in Zang *et al.* [26]. Aside from the light source, the flicker ERG was conducted using the same set-up as the white light ERG. Flicker frequencies of 5, 8, 10 and 15 Hz with a 50% duty cycle were applied. The response power at each frequency was analysed using MATLAB (R2023b). Figures were generated, and statistical analyses were performed with GraphPad Prism 9 (GraphPad Software, San Diego, CA, USA).

## Data Availability

All data and reagents are freely available upon request.
